# Chyme reinfusion via percutaneous endoscopic colostomy in type 1 short bowel syndrome with a defunctionalized but intact colon: short-term nutritional outcomes and parenteral support weaning

**DOI:** 10.3389/fnut.2026.1878745

**Published:** 2026-09-21

**Authors:** Jingfei Sun, Xin Xu, Risheng Zhao, Zheng Yao, Yunzhao Zhao, Qian Huang

**Affiliations:** 1 Department of General Surgery, Jiangning Hospital, Nanjing, Jiangsu, China; 2 Department of General Surgery, Jinling Hospital, Nanjing, Jiangsu, China

**Keywords:** chyme reinfusion, nutritional status, parenteral nutrition, percutaneous endoscopic colostomy, short bowel syndrome

## Abstract

**Background:**

Short bowel syndrome (SBS) type I, characterized by an end stoma without colonic continuity, poses significant challenges in fluid and electrolyte management. The defunctionalized colon remains dormant before stoma reversal, representing an untapped absorptive reserve. Percutaneous endoscopic colostomy (PEC) provides a minimally invasive route for scheduled chyme reinfusion, yet evidence regarding its nutritional benefits remains limited. This study aimed to examine whether PEC-guided colonic chyme reinfusion before stoma reversal is associated with improved nutritional outcomes in type I SBS patients with preserved colon.

**Methods:**

This retrospective cohort study included 71 type I SBS patients with retained intact colon undergoing stoma reversal between August 2019 and May 2024. Patients were divided into the PEC group (n = 57, treated after 2021) and the non-PEC historical control group (n = 14, treated before 2021). Nutritional outcomes, including serum albumin, prealbumin, C-reactive protein (CRP), body mass index (BMI), electrolyte supplementation requirements, intravenous fluid requirements, and enteral nutrition tolerance, were assessed at admission and at 2 months after admission. Linear mixed-effects models and logistic regression were used for statistical analysis.

**Results:**

Both groups showed significant improvements in most nutritional parameters over time. The PEC group demonstrated significantly greater reductions in intravenous fluid requirements (1761 vs. 3305 mL, *P* < 0.0001), sodium supplementation (3.91 vs. 7.63 g, *P* < 0.0001), and potassium supplementation (2.50 vs. 4.73 g, *P* < 0.0001) at 2 months. Enteral nutrition tolerance improved more substantially in the PEC group (51.2 vs. 40.0 mL/h, *P* < 0.0001). Prealbumin levels increased significantly more in the PEC group, with a significant time-by-group interaction (*P* = 0.014). PEC-related complications occurred in 4 patients (7.0%), all Grade I or II. However, no significant between-group differences were observed in CONUT and NRS2002 scores, albumin, or BMI at the 2-month time point.

**Discussion:**

PEC-guided chyme reinfusion in type I SBS patients with a defunctionalized but intact colon was associated with reduced parenteral fluid and electrolyte requirements, improved enteral tolerance, and increased prealbumin levels within a 2-month preoperative window, with an acceptable safety profile. Global nutritional screening scores showed limited changes, suggesting that the early benefits of PEC are primarily manifested in reduced parenteral support dependence rather than broad nutritional indices. Prospective randomized trials with longer follow-up are warranted to confirm these findings.

## 1 Introduction

Short bowel syndrome (SBS) is a severe malabsorptive condition often requiring long-term parenteral support (1). Anatomic configuration critically determines outcome: in Type I SBS (end stoma, no colonic continuity), the minimal small bowel length required for enteral autonomy is approximately 100 cm, whereas in Type II/III SBS with preserved colonic continuity, this threshold drops to 50–60 cm—owing to the colon's ability to absorb fluid, electrolytes, and short-chain fatty acids (2, 3). This physiological principle implies that even in Type I patients with an anatomically intact but defunctionalized colon, restoring luminal flow could unlock colonic absorptive reserve and reduce the effective small bowel length demand.

Clinically, stoma reversal is the definitive treatment to restore continuity, yet it is typically delayed until patients achieve metabolic stability to minimize surgical risks (4). This precaution, however, leaves the colon dormant for prolonged periods, potentially missing a critical window for adaptation. Current preoperative management remains largely passive—focusing on fluid/electrolyte replacement, pharmacological reduction of stoma output, and gradual enteral feeding advancement—without actively harnessing the absorptive capacity of the defunctionalized colon. Prolonged colonic diversion may lead to mucosal atrophy and reduced short-chain fatty acid production, compromising the colon's contribution to nutritional recovery once continuity is restored. Consequently, patients often remain dependent on parenteral support, with attendant risks of catheter-related infections, progressive liver injury, and impaired quality of life.

In patients with an end jejunostomy, these challenges are compounded by the jejunum's distinct physiology. Unlike the ileum, the jejunum lacks the “ileal brake”—a feedback mechanism that slows transit in response to distal nutrients—resulting in rapid flow and shortened absorption time (5). Moreover, the jejunal epithelium has a highly permeable paracellular pathway and limited sodium absorptive capacity. When oral intake is hypotonic, rapid water absorption creates a luminal sodium gradient that drives passive sodium loss into the gut lumen, paradoxically worsening net sodium depletion (6). Hypertonic intake, conversely, draws water into the lumen, further increasing stoma output. These osmotic forces, combined with rapid transit, make Type I SBS patients particularly vulnerable to fluid and sodium depletion, underscoring the physiological rationale for restoring colonic exposure to luminal chyme.

It is reasonable to hypothesize that early chyme reinfusion into the defunctionalized colon could serve as a biological bridge to precondition the colon and improve preoperative nutritional status (7–9). Percutaneous endoscopic colostomy (PEC), originally developed for colonic decompression (10, 11), provides a minimally invasive and reproducible access route for scheduled reinfusion—yet evidence on its nutritional benefits in SBS remains scarce. In this exploratory retrospective study, we aim to examine whether PEC-guided colonic chyme reinfusion before stoma reversal is associated with improved nutritional outcomes in Type I SBS patients with preserved colon.

## 2 Materials and methods

### 2.1 Treatment for SBS with preserved intact defunctionalized colon

#### 2.1.1 Standardized treatment protocol

The standardized protocol comprised three components. (1) Fluid and electrolyte management: Laboratory tests were performed every 2–3 days to guide electrolyte replacement. Daily body weight, urine output (target ≥1 L/day), and stoma output were recorded for fluid calculation (12). (2) Nutritional support: Oral intake was withheld preoperatively. Initial parenteral nutrition (PN) provided 1.5 times resting energy expenditure (13). After hemodynamic stabilization, enteral nutrition (EN) via nasogastric tube was introduced and advanced according to stoma output tolerance, with the goal of achieving PN independence while maintaining weight and nutrition solely via EN. (3) Pharmacotherapy: Initial regimen included omeprazole (gastric acid suppression) and somatostatin (6 mg/day) to reduce intestinal fluid losses; somatostatin was later adjusted based on stoma output variability. Montmorillonite powder and loperamide were used routinely.

#### 2.1.2 PEC placement and reinfusion protocol

PEC protocol was not performed until beginning of 2021; thus, no patients received PEC prior to that year. Thereafter, PEC was offered to eligible patients with anatomically intact colon, absence of colonic obstruction/fistula, and no endoscopic evidence of ulceration, ischemia, or mucosal erosion. A colonoscope was advanced to the cecum/ascending colon. The optimal puncture site was identified via transillumination and finger indentation, marked, sterilized, and anesthetized. Using a 20Fr PEG kit, a needle was inserted percutaneously into the colonic lumen under endoscopic guidance. A guidewire was passed through the needle, grasped with a snare via the colonoscope, and withdrawn through the anus. The PEC tube was tied to the wire, a small skin incision made, and the tube pulled retrograde through the colon and out the abdominal wall. The tube was shortened, secured with an external bumper, and correct placement confirmed by repeat colonoscopy. Following PEC placement and a five-day observation period without significant puncture site exudate, computed tomography and contrast studies confirmed proper catheter positioning (intraluminal tip without obstruction/kinking) and the absence of intra-abdominal fluid. Catheter patency was maintained with regular saline flushes (10 mL every 8 hours). In cases of suspected catheter occlusion, gentle flushing with warm saline was attempted; if unsuccessful, endoscopic revision was considered. Saline infusion began at 20 mL/hour. After one week, gradual transition to chyme reinfusion commenced, progressing to full reinfusion (all stoma output returned). Chyme collected from the ostomy bag was reinfused four times daily (6:00, 12:00, 18:00, 24:00) using an EN pump. The strategy prioritized achieving full chyme reinfusion before aggressively advancing EN, as increased EN typically raised stoma output and reinfusion pressure.

#### 2.1.3 Stoma reversal criteria

Stoma reversal was recommended when patients met all of the following criteria: stable albumin, adequate physical strength, Body Mass Index(BMI) >18.5 kg/m^2^, ≥2 months post-admission, and sustained full chyme reinfusion for ≥1 month (14). For non-PEC patients, criteria were identical except requiring stoma output < 1000 mL/day (with pharmacological support) instead of chyme reinfusion.

### 2.2 Patients and follow-up

In this retrospective cohort study, type 1 SBS was defined as residual small intestine length < 200 cm proximal to the stoma (1–3). Patients were referred from external hospitals. Included were those undergoing stoma reversal for type 1 SBS with retained intact colon between August 2019 and May 2024. Exclusion criterion: incomplete clinical records. According to the timeline for the implementation of PEC (PEC was not implemented from 2019 to 2020; it was widely implemented after 2021), patients were divided into non-PEC group and PEC group.

As reversal was considered after 2 months of admission, we followed patients up to this time point to assess the impact of PEC reinfusion on preoperative nutritional status. The outcomes included nutritional screening scores, biochemical nutritional markers, parenteral support requirements, enteral tolerance, and complications—including PEC-related complications, refeeding syndrome, and parenteral nutrition-associated liver disease (PNALD). Because this retrospective study lacked direct measurements of body composition and muscle mass, Controlling Nutritional Status Score (CONUT) and NRS (Nutrition Risk Screening) 2002 were used as screening tools to complement laboratory and clinical indicators.

### 2.3 Data collection and statistical analysis

Data were extracted from electronic medical records using a standardized case report form. Baseline demographic and clinical characteristics included age, sex, body mass index (BMI), etiology of SBS, residual small bowel length, preserved colon length, stoma output status, and time from SBS diagnosis to PEC placement. Laboratory parameters—including albumin, prealbumin, C-reactive protein (CRP), serum sodium, and serum potassium—were measured at admission and at 2 months post-admission. Nutritional screening scores (CONUT and NRS2002) were calculated at both time points using standard formulas. Intravenous fluid and electrolyte supplementation requirements, as well as enteral nutrition tolerance, were recorded daily.

Statistical methods: Continuous variables are presented as medians with interquartile ranges (IQR), and categorical variables as frequencies with percentages (%). Between-group comparisons at baseline were performed using the Mann–Whitney U test for continuous variables and the chi-square test or Fisher's exact test for categorical variables, as appropriate.

For longitudinal outcomes (CONUT, NRS2002, albumin, prealbumin, CRP, BMI, sodium supplementation, potassium supplementation, intravenous fluid requirement, and enteral nutrition rate), linear mixed-effects models were employed. Each model included time, group, and their interaction as fixed effects, adjusting for the following prespecified covariates: etiology, residual small bowel length, preserved colon length, and high-output stoma status. Linear mixed-effects models were selected because they accommodate the inherent correlation structure of repeated measurements, handle unbalanced data robustly, and allow simultaneous modeling of both within-subject changes over time and between-subject differences. Pairwise comparisons between and within groups were performed using estimated marginal means with appropriate contrasts from the fitted models.

Logistic regression was used to assess the association between PEC reinfusion and two binary outcomes: (1) CONUT downgrading, defined as a reduction to a lower risk category (from moderate/severe to mild/normal) at 2 months; and (2) NRS2002 risk resolution, defined as achieving NRS2002 ≤ 3 at 2 months. Both unadjusted and adjusted models were fitted.

Handling of missing data: No imputation was performed for missing data. Patients with incomplete clinical records were excluded from the study during the screening process. For the longitudinal outcomes, the linear mixed-effects models used all available measurements at each time point and do not require complete case records, as these models are robust to intermittent missing data under the missing-at-random assumption.

This study was retrospective in design, and all analyses were exploratory in nature. No formal analysis plan was pre-registered or pre-specified prior to data collection. The primary outcomes—including changes in nutritional screening scores (CONUT, NRS2002), biochemical markers (albumin, prealbumin), and parenteral support requirements (IV fluid, sodium, potassium)—were defined before conducting the statistical analyses based on the study hypothesis that PEC-guided reinfusion would improve nutritional status and reduce dependence on parenteral support. All findings should be interpreted as hypothesis-generating rather than confirmatory. Given the retrospective and exploratory nature of this study, no formal sample size calculation was performed *a priori*. The sample represents the complete cohort of eligible patients treated at our institution during the study period who met the inclusion criteria. As is typical for exploratory studies, we included all available eligible cases to maximize statistical power within the constraints of a single-center retrospective design.

Complication reporting: In the PEC subgroup, PEC-related complications were classified according to the Clavien–Dindo classification system (15) and reported descriptively. No statistical comparison was performed for complications given the small number of events. Refeeding syndrome was diagnosed if (1) at least one of serum phosphorus, potassium, or magnesium levels decreased by >10% from baseline (16). For exploratory analysis of metabolic safety, PNALD was defined as elevation of at least two of the following enzymes to >1.5 × the upper limit of normal (ULN) at 2 weeks: ALT, AST, and ALP (17).

All statistical analyses were performed using R version 4.4.3 (R Foundation for Statistical Computing, Vienna, Austria) and SPSS version 26.0 (IBM Corp., Armonk, NY, USA). A two-sided P < 0.05 was considered statistically significant for all analyses.

## 3 Results

### 3.1 Patient characteristics

A total of 71 patients were included after excluding 3 with incomplete data: 57 in the PEC group and 14 in the non-PEC group (Figure 1). All 14 non-PEC patients were not scheduled for the procedure before 2021. Of the 57 patients in PEC group, the median time from SBS diagnosis to PEC placement was 32 days (IQR: 23-43). Full chyme reinfusion was achieved at a median of 14 days (IQR: 11-23) post-PEC. Baseline characteristics are summarized in Table 1. Most patients were male (*n* = 41, 57.7%). Median age was 47 years (IQR: 34-56), and median BMI was 19.5 kg/m^2^ (IQR: 17.7-22.1). Median residual small bowel length proximal to the stoma was 130 cm (IQR: 80-150). A stoma output ≥1000 mL/day was present in 53 patients (74.6%). The most common etiology was radiation-induced intestinal injury (*n* = 37, 52.1%), followed by mesenteric thrombosis (*n* = 18, 25.4%) and trauma (*n* = 12, 16.9%). No significant baseline differences were observed between the two groups.

**Figure 1:**
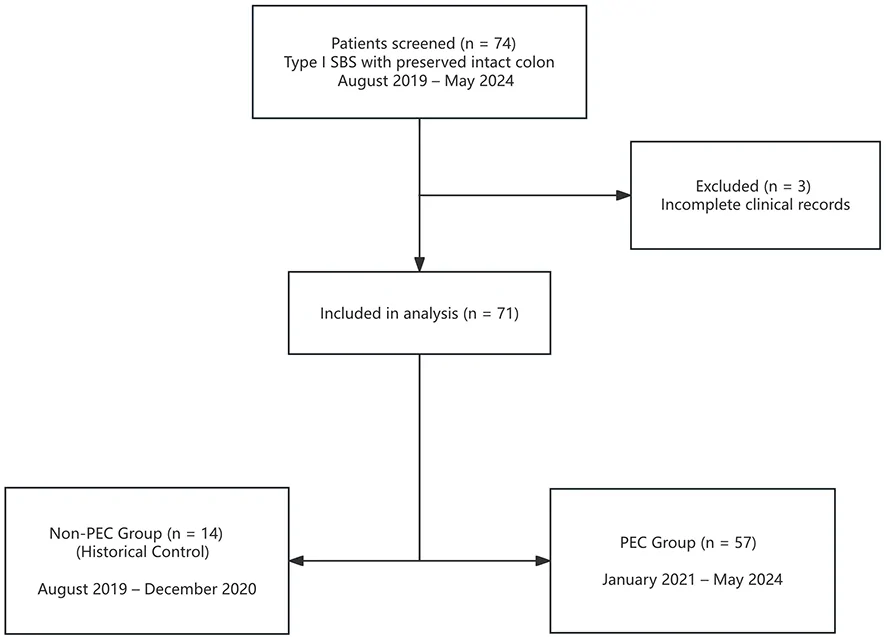
Of 74 patients screened, three were excluded due to incomplete records. The remaining 71 patients were allocated to the non-PEC group (*n* = 14) and PEC group (*n* = 57).

**Table 1:** Characteristics of the enrolled 71 patients at admission.

Characteristics	Total cohort	PEC	Non-PEC	*P*
Demographic data				
Male,No. (%)	41 (57.7)	33 (57.9)	8 (57.1)	0.96
Age, years;(median, IQR)	47 (34–56)	47 (34–56)	49 (42–57)	0.57
BMI, kg/m^2^, (median, IQR)	19.5 (17.7–22.1)	19.4 (17.7–22.6)	19.8 (17.7–20.4)	0.82
SBS characteristics at admission				
Previous abdominal surgery times, *n*, (median, IQR)	2(1–3)	2(1–3)	2(1–3)	0.67
Resting energy expenditure, REE, Kcal/day, (median, IQR)	1270 (1159 – 1393)	1278 (1157 – 1367)	1257 (1168 – 1433)	0.63
Length of small bowel proximal to stoma, cm (median, IQR)	150 (100 – 180)	150 (100 – 180)	150 (100–180)	0.66
Length of small bowel proximal to stoma, No. (%)				0.61
< 100cm	19 (26.8)	16 (10.5)	3 (21.4)	
≥ 100cm	52 (73.2)	41 (71.9)	11 (78.6)	
Length of small bowel distal to stoma, No. (%)				0.23
≤ 20cm	66 (92.9)	54 (94.7)	12 (85.7)	
> 20cm	5 (7.1)	3 (5.3)	2 (14.3)	
Stoma fluid volume ≥ 1000 mL^*^, No. (%)	39 (54.9)	31 (54.4)	8 (57.1)	0.85
Enteral nutrition feeding rate, ml/h, (median, IQR)	20 (0 – 20)	15 (0 – 20)	20 (0 – 20)	0.21
Colon length, cm, (median, IQR)	100 (90 – 110)	100 (90 – 110)	100 (90 – 105)	0.81
Interval from diagnosed SBR to PEC, days, (median, IQR)	32 (23 – 43)	32 (23 – 43)	38 (24 – 52)	0.24
Etiology, No. (%)				0.65
Trauma	12 (16.9)	10 (17.5)	2 (14.3)	
Mesenteric thrombosis	18 (25.4)	15 (26.3)	3 (21.4)	
Radiation-induced intestinal injury	37 (52.1)	28 (49.1)	9 (64.3)	
Inflammatory bowel disease	4 (5.6)	4 (7.1)	0 (0)	
Daily intravenous fluid replacement,ML, (median, IQR)	4300 (4000–4900)	4300 (4000 – 4900)	4400 (4000– 4900)	0.60
Laboratory test results at admission				
Hemoglobin, g/L; (median, IQR)	93 (80 – 106)	97 (80 – 103)	92 (79 – 107)	0.97
Albumin, g/L; (median, IQR)	30 (26 – 33)	30 (26 – 34)	31 (27 – 33)	0.43
Prealbumin, g/L; (median, IQR)	17 (15 – 21)	17 (15 – 21)	18 (14 – 22)	0.84
Alanine Aminotransferase, U/L; (median, IQR)	30 (17 – 46)	31 (17 – 47)	29 (13 – 39)	0.34
Aspartate Aminotransferase, U/L; (median, IQR)	46 (29 – 62)	46 (29 – 60)	48 (31 - 67)	0.70
Gamma-Glutamyl Transferase, U/L; (median, IQR)	102 (69 – 122)	102 (66 – 121)	100 (78 – 132)	0.56
Alkaline phosphatase, U/L; (median, IQR)	69 (42 – 104)	68 (42 – 99)	81 (41 – 126)	0.36
Bilirubin, umol/L; (median, IQR)	21 (15 – 25)	21 (15 – 26)	20 (13 – 24)	0.72
Comorbidity				
Chronic hepatitis, No. (%)	1 (1.4)	1 (1.7)	0	0.62
Chronic renal insufficiency, No. (%)	0	0	0	1.00
Diabetes, No. (%)	1 (1.4)	1 (1.7)	0	0.62

^*^the somatostatin was used in all the patients at admission.

### 3.2 Changes in nutritional screening score

The median CONUT of the 71 patients at admission was 5 (IQR:4 - 6). There were no significant difference between patients with or without PEC(5[IQR:4 - 6] vs. 5[IQR:4 - 6], *P* = 0.95). After adjusting for residual small bowel length, etiology, age, and output status, linear mixed-effects modeling revealed a significant main effect of time on CONUT scores (*F* = 96.72, *P* < 0.0001), but no significant main effect of group (*F* = 0.45, *P* = 0.505) nor a time-by-group interaction (F = 1.19, *P* = 0.279). Residual small bowel length ≥100 cm was independently associated with better nutritional status (*F* = 5.81, *P* = 0.019). Both groups showed significant CONUT reductions from admission to 2 months (PEC: −3.04, *p* < 0.0001; non-PEC: −2.43, *P* < 0.0001;linear mixed-effects model with pairwise comparisons). However, no significant between-group differences were observed at endpoint (*P* = 0.22, Figure 2).

**Figure 2:**
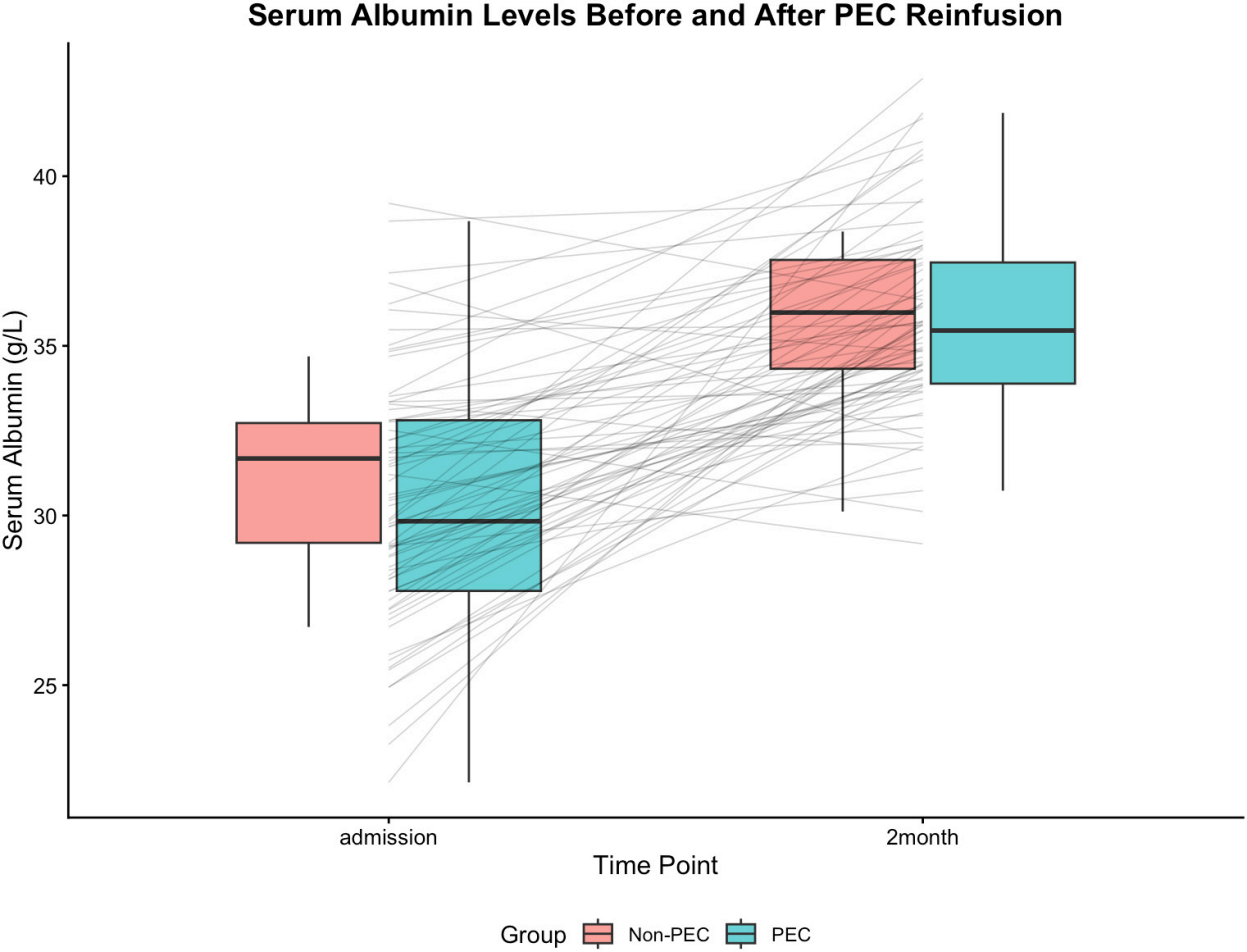
CONUT score trajectories over 2 months in PEC vs. non-PEC groups. Both groups showed significant improvement over time (*P* < 0.0001), with no between-group difference at 2 months (*P* = 0.22).

According to the CONUT, the risks were classified into normal (0–1), light (2–4), moderate (5–8), and severe (9–12). Different levels of personnel distribution were revealed in Table 2, without difference between groups (*P* = 0.74). We conducted a logistic regression analysis and found that PCE reinfusion after 2 months of treatment could not downgrade CONUT (unadjusted *OR* = 2.54, 95%*CI*: 0.76 – 8.66, *P* = 0.14).

**Table 2:** Personnel distribution according to CONUT score.

Severity according to CONUT score	PEC	Non-PEC	*P*
			0.64
Normal (0–1)	0	0	
Light(2–4)	21	4	
Moderate (5–8)	34	9	
Severe (9–12)	2	1	

The NRS2002 score at admission was five (*IQR*:5–6) of the total cohort. There were no significant difference between groups (*P* = 0.95). All the patients were in high malnutrition risk (NRS2002 >3) at admission. After the 2 months of admission, the NRS2002 score still >3 occurred in 26 patients (36.1%). The unadjusted regression revealed that reinfusion via PEC could not alleviate the risk of malnutrition (*OR* = 0.36, 95%*CI*:0.11 – 1.15, *P* = 0.08). We then adjusted the factors with unadjusted regression *P* < 0.1 less than including length of small bowel proximal to stoma, and output of stoma, revealing the similarly results (*OR* = 0.43;95%*CI*:0.12 – 1.71; *P* = 0.19, Figure 3).

**Figure 3:**
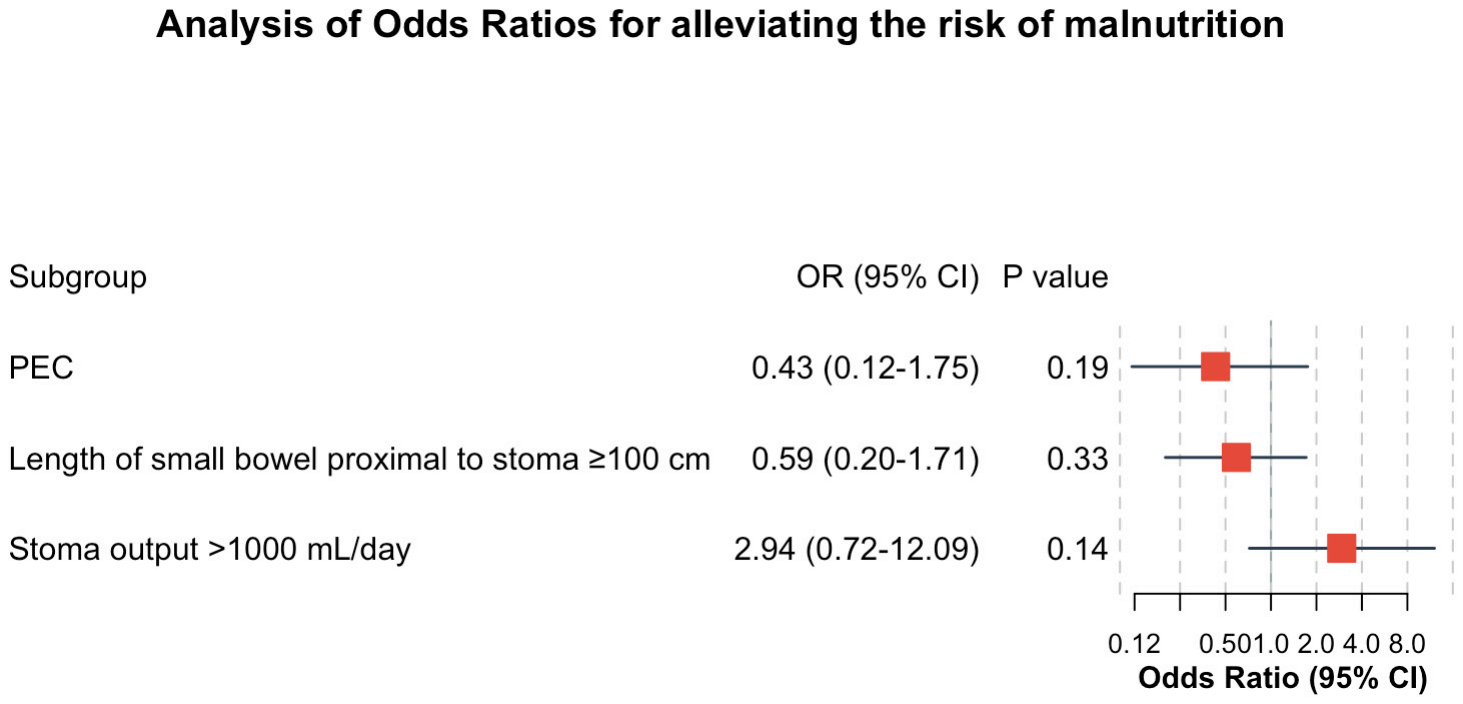
Forest plot of the adjusted association between PEC-guided chyme reinfusion and NRS2002-defined malnutrition risk resolution at 2 months.

### 3.3 Longitudinal changes in nutritional and support-related outcomes

Linear mixed-effects models were fitted to evaluate changes from admission to 2 months after admission for eight outcomes, adjusting for etiology, residual small bowel length, colon length, and output stoma status (Table 3). All outcomes showed significant improvement over time (all *P* < 0.001), except for the time effect on BMI (Figure 4).

**Table 3:** Linear mixed-effects models fitted to evaluate nutritional and clinical outcomes changes from admission to 2 months after admission adjusting for etiology, residual small bowel length, colon length, and output stoma status.

Outcome	PEC group change	Non-PEC group change	Difference-in-Difference	*P* for interaction	*P* for 2-month between-group difference
Albumin (g/L)	+5.64^***^	+3.87^***^	+1.77	0.181	0.972
Prealbumin (mg/L)	+13.0^***^	+10.3^***^	+2.7	0.014	0.021
CRP (mg/L)	24.5^***^	−19.4^***^	−5.1	0.431	0.622
BMI (kg/m^2^)	+2.57^***^	+1.34	+1.23	0.175	0.056
Sodium supplement (g)	−6.12^***^	−2.29^***^	−3.83	< 0.0001	< 0.0001
Potassium supplement (g)	−4.32^***^	−2.29^***^	−1.96	< 0.0001	< 0.0001
IV fluid (mL)	−2695^***^	−1229^***^	−1466	< 0.0001	< 0.0001
Enteral nutrition (mL/h)	+37.5^***^	+23.6^***^	+13.9	< 0.0001	< 0.0001

^***^P < 0.001 for within-group change from baseline; All changes are adjusted mean differences from linear mixed-effects models.

**Figure 4:**
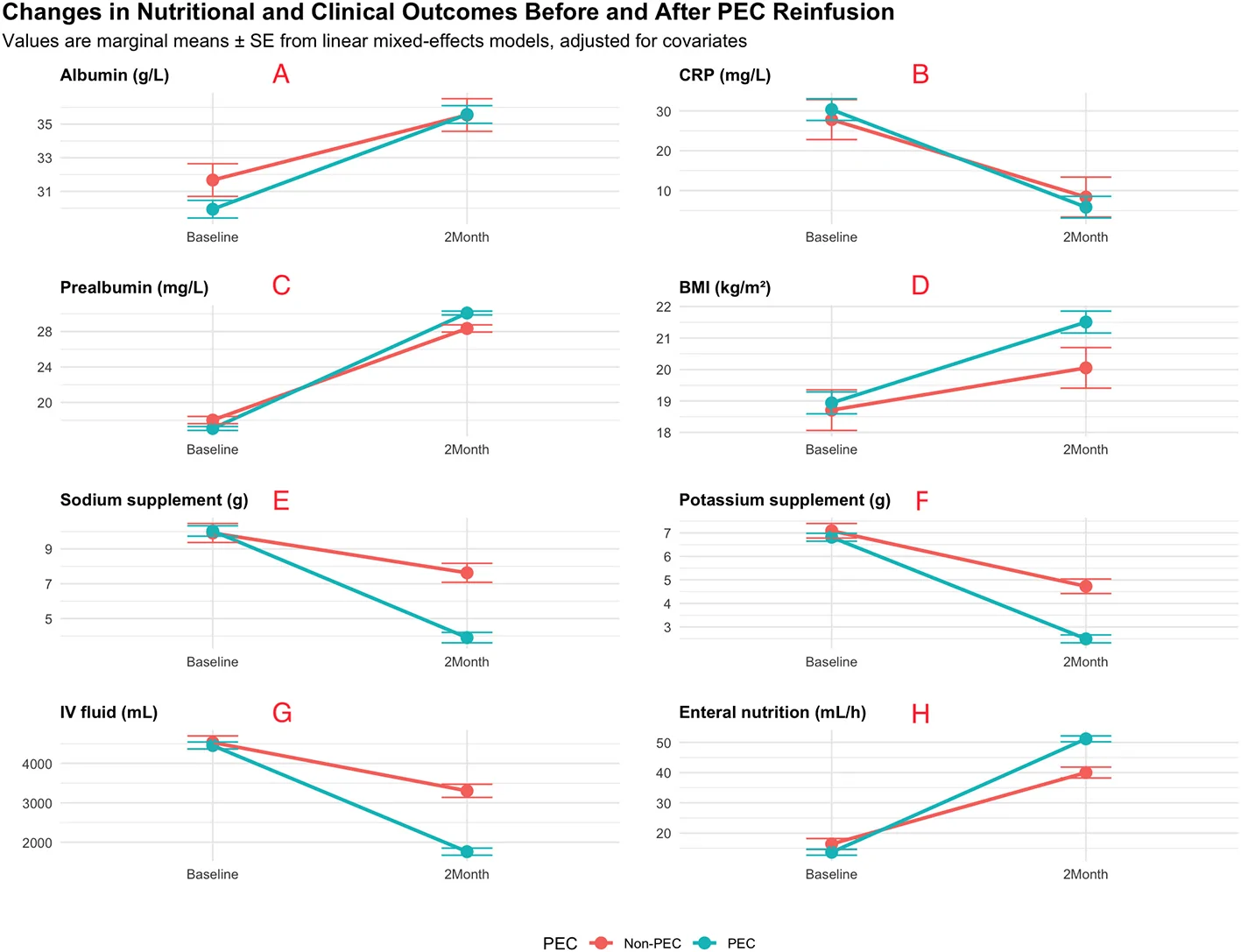
Longitudinal changes in nutritional and clinical outcomes from admission to 2 months after admission. Panels show trajectories for eight outcomes: **(A)** serum albumin (g/L), **(B)** C-reactive protein (CRP, mg/L), **(C)** prealbumin (mg/L), **(D)** body mass index (BMI, kg/m^2^), **(E)** sodium supplementation (g/day), **(F)** potassium supplementation (g/day), **(G)** intravenous (IV) fluid requirements (mL/day), and **(H)** enteral nutrition (EN) rate (mL/h). Data are presented as medians with interquartile ranges (IQR). Linear mixed-effects models were fitted adjusting for etiology, residual small bowel length, colon length, and high-output stoma status. All outcomes showed significant improvement over time (all *P* < 0.001), except for BMI. Significant time-by-group interactions favoring the PEC group were observed for prealbumin (*F* = 15.31, *P* = 0.014), sodium supplementation (*P* < 0.0001), potassium supplementation (*P* < 0.0001), IV fluid requirements (*P* < 0.0001), and EN rate (*P* < 0.0001). At 2 months, the PEC group had significantly higher prealbumin levels (30.08 vs. 28.35 mg/L; *P* = 0.021), lower sodium (3.91 vs. 7.63 g/day; *P* < 0.0001) and potassium requirements (2.50 vs. 4.73 g/day; *P* < 0.0001), reduced IV fluid needs (1761 vs. 3305 mL/day; *P* < 0.0001), and higher EN tolerance (51.2 vs. 40.0 mL/h; *P* < 0.0001) compared with the non-PEC group. No significant between-group differences were observed for albumin, CRP, or BMI at 2 months. PEC, between groups.

#### 3.3.1 Albumin

Both groups demonstrated significant increases in serum albumin from admission to 2 months (PEC: +5.64 g/L, *P* < 0.0001; non-PEC: +3.87 g/L, *P* = 0.0015). However, the time-by-group interaction was not significant (*P* = 0.181), and no between-group difference was observed at 2 months (difference: −0.03 g/L, 95% *CI*: −1.99 to 1.92, *P* = 0.972). Colon length was independently associated with albumin levels (*F* = 4.15, *P* = 0.046).

#### 3.3.2 C-Reactive Protein

CRP levels decreased markedly in both groups (PEC: −24.5 mg/L, *P* < 0.0001; non-PEC: −19.4 mg/L, *P* = 0.0011), with no significant interaction (*P* = 0.431) or between-group difference at 2 months (difference: +2.53 mg/L, 95% CI: −7.51 to 12.57, *P* = 0.622).

#### 3.3.3 Prealbumin

Prealbumin increased substantially in both groups (PEC: +13.0 mg/L, *P* < 0.0001; non-PEC: +10.3 mg/L, *P* < 0.0001). Notably, a significant time-by-group interaction was observed (*F* = 15.31, *P* = 0.014), indicating that the PEC group achieved a greater magnitude of improvement. At 2 months, the PEC group had significantly higher prealbumin levels compared with the non-PEC group (30.08 vs. 28.35 mg/L; difference: −1.73 mg/L, 95% *CI*: −2.56 to −0.92, *P* = 0.021).

#### 3.3.4 Body Mass Index

BMI increased significantly in the PEC group (+2.57 kg/m^2^, *P* < 0.0001), while the increase in the non-PEC group did not reach statistical significance (+1.34 kg/m^2^, *P* = 0.099). The time-by-group interaction was not significant (*P* = 0.175). At 2 months, the PEC group had comparable BMI compared with the non-PEC group (21.51 vs. 20.08 kg/m^2^; difference: −1.43 kg/m^2^, 95% *CI*: −2.57 to −0.17, *P* = 0.056).

#### 3.3.5 Sodium supplement

Sodium supplementation requirements decreased significantly in both groups (PEC: −6.12 g, *P* < 0.0001; non-PEC: −2.29 g, *P* < 0.0001), with a significant interaction (*P* < 0.0001) favoring the PEC group. At 2 months, the PEC group required substantially less sodium supplementation compared with the non-PEC group (3.91 vs. 7.63 g; difference: 3.72 g, 95% *CI*: 2.64 to 4.80, *P* < 0.0001).

#### 3.3.6 Potassium supplement

Similar to sodium, potassium requirements decreased significantly in both groups (PEC: −4.32 g, p < 0.0001; non-PEC: −2.36 g, p < 0.0001), with a significant interaction (p < 0.0001). At 2 months, the PEC group required significantly less potassium supplementation (2.50 vs. 4.73 g; difference: 2.23 g, 95% *CI*: 1.61 to 2.85, p < 0.0001).

#### 3.3.7 Intravenous fluid requirements

IV fluid requirements decreased dramatically in both groups (PEC: −2695 mL, *P* < 0.0001; non-PEC: −1229 mL, *P* < 0.0001), with a significant interaction (*P* < 0.0001). At 2 months, the PEC group required significantly less IV fluid (1761 vs. 3305 mL; difference: 1545 mL, 95% *CI*: 1209 to 1880, *P* < 0.0001). Etiology was also independently associated with IV fluid requirements (*F* = 3.19, *P* = 0.029).

#### 3.3.8 Enteral nutrition

EN tolerance improved significantly in both groups (PEC: +37.5 mL/h, *P* < 0.0001; non-PEC: +23.6 mL/h, *P* < 0.0001), with a significant interaction (*P* < 0.0001). At 2 months, the PEC group achieved significantly higher EN rates (51.2 vs. 40.0 mL/h; difference: −11.2 mL/h, 95% CI: −14.8 to −7.5, *P* < 0.0001).

### 3.4 . Complications

PEC-related complications occurred in four patients (7.0%). All complications were Grade I or II and occurred after reinfusion, including gastrointestinal bleeding (*n* = 3; 2 Grade I, 1 Grade II) and puncture site infection (*n* = 1, Grade I). PNALD was identified in 19 patients (16 in PEC group vs. three in non-PEC group; *P* = 0.62). Refeeding syndrome was observed in 52 patients (42 in PEC group vs. 10 in non-PEC group; *P* = 0.87).The incidence of Refeeding syndrome was significantly lower in patients with small bowel length ≥ 1 m compared with those < 1 m (25/40 vs. 27/31; *P* = 0.02). Regarding colonic symptoms after reversal in the non-PEC group, five of 14 patients (35.7%) experienced diarrhea during the postoperative period, among whom one presented with minor colonic bleeding that resolved with conservative management. However, because routine colonoscopy was not performed prior to stoma reversal, the presence of diversion colitis could not be systematically confirmed or excluded. No such symptoms were documented in the PEC group.

## 4 Discussion

In this retrospective cohort study of 71 patients with type I short bowel syndrome and a preserved but defunctionalized colon, PEC-guided chyme reinfusion was associated with several clinically meaningful short-term benefits before stoma reversal. Compared with conventional management, PEC significantly reduced intravenous fluid, sodium, and potassium requirements, improved enteral nutrition tolerance, and increased prealbumin levels. These findings suggest that PEC may serve as a feasible bridge therapy for preoperative optimization in selected patients with type I SBS (1, 3, 12, 14, 18).

The most consistent benefit observed in this study was the reduction in parenteral support dependence. Type I SBS, defined by an end stoma without colonic continuity, is a particularly challenging form of intestinal failure because the colon cannot participate in fluid and electrolyte salvage (1, 3, 12). In contrast, patients with colon-in-continuity generally have a lower requirement for parenteral support, reflecting the colon's ability to absorb water, sodium, and short-chain fatty acids (5, 19, 20). Restoring chyme delivery to the defunctionalized colon through PEC may therefore reactivate colonic absorptive function and reduce gastrointestinal losses. This mechanism is consistent with our findings that the PEC group required substantially less intravenous fluid and electrolyte supplementation at 2 months. From a practical standpoint, this benefit is highly relevant because excessive parenteral support increases treatment burden and may contribute to catheter-related complications.

Another important finding was the improvement in enteral tolerance. The PEC group achieved higher enteral nutrition rates at follow-up, suggesting that chyme reinfusion may facilitate intestinal adaptation and allow more effective advancement of enteral feeding. Prior studies of chyme reinfusion in patients with temporary enterostomies or intestinal failure have similarly shown improvements in nutritional balance and support reduction (7, 14, 19). Although the exact biological pathways remain incompletely understood, reinfused chyme may stimulate mucosal adaptation, preserve luminal signaling, and enhance colonic short-chain fatty acid production (7, 21). This may be particularly important in patients awaiting stoma reversal, in whom gradual advancement of enteral feeding is often limited by high output and fluid loss.

Prealbumin also improved more in the PEC group, indicating a potential advantage in short-term protein nutritional status. Compared with albumin, prealbumin has a shorter half-life and is more sensitive to recent changes in nutritional intake and metabolic balance. The observed improvement may reflect the combined effects of reduced fluid loss, better enteral tolerance, and improved nutritional delivery during the reinfusion period. This interpretation is compatible with the concept that restoration of colonic continuity can improve metabolic efficiency in SBS patients, particularly when the colon remains anatomically intact (3, 7, 18).

By contrast, changes in CONUT, NRS2002, albumin, and BMI were less pronounced. These measures may not fully capture the short-term physiological effects of chyme reinfusion in this setting. CONUT and NRS2002 are broad nutritional screening tools and may be less sensitive to rapid changes in fluid balance, electrolyte homeostasis, or enteral adaptation (1, 12). Albumin and BMI also tend to change slowly and are influenced by inflammation, hydration status, and the overall catabolic burden of illness. Therefore, limited changes in these indices should not be interpreted as absence of benefit, but rather as evidence that PEC exerts its earliest and most measurable effects on parenteral support dependence and enteral tolerance.

The physiological basis for these findings is consistent with the known function of the colon. The colon plays an important role in water and sodium absorption and in energy salvage through short-chain fatty acids derived from microbial fermentation (5, 21–23). However, its contribution to protein synthesis and global nutritional recovery is limited. Accordingly, chyme reinfusion would be expected to produce early benefits in fluid and electrolyte handling, while broader nutritional indices may require longer follow-up or more substantial small bowel adaptation. This may also explain why the most robust differences in our study were observed in support-related outcomes rather than in composite screening scores.

The finding that colon length was associated with better albumin outcomes further supports the importance of colonic absorptive reserve. A longer colon may provide greater mucosal surface area and improved capacity for fluid and nutrient salvage, which could enhance the benefits of reinfusion. This aligns with prior work emphasizing the role of the colon in SBS outcomes and nutritional autonomy (7, 13, 18). However, because segmental colonic anatomy and detailed mucosal status were not available in our study, future studies should investigate whether specific colonic regions contribute differentially to the response to chyme reinfusion.

The safety profile of PEC in this cohort was acceptable. Complications occurred in 7.0% of patients and were all mild (Clavien–Dindo Grade I or II), including gastrointestinal bleeding (*n* = 3) and puncture site infection (*n* = 1), all managed conservatively. PEC was originally developed as a minimally invasive technique for colonic decompression in conditions such as Ogilvie syndrome and sigmoid volvulus (10, 11). Our findings suggest that this technique can also be repurposed safely as an access route for scheduled chyme reinfusion in SBS patients. Similar safety signals have been reported in prior chyme reinfusion cohorts, supporting the feasibility of this approach (7, 14, 19). No cases of refeeding syndrome were observed during enteral nutrition advancement or chyme reinfusion in either group. Longitudinal changes in liver function parameters did not differ significantly between the two groups over the 2-month observation period.

Beyond safety, several practical considerations merit discussion before PEC-guided reinfusion can be integrated into routine practice. First, the procedure requires experienced interventional endoscopists and close collaboration with nutritional support teams; the learning curve should not be underestimated. Second, although patient acceptance and quality of life were not formally assessed in this retrospective study, we acknowledge that daily reinfusion and tube care may impose a substantial burden. Third, the reinfusion protocol increases nursing workload; strategies to mitigate this burden may include simplifying the infusion schedule, using standardized sets to streamline preparation, and establishing clear clinical pathways. Fourth, logistical challenges, including chyme collection, handling, and contamination risk, are manageable in hospitalized settings but require adaptation for outpatient programs, such as closed collection systems, caregiver training, and telehealth support. The conventional management used in the non-PEC group may also have contributed to the relatively favorable outcomes observed in both groups. Standard treatment for high-output intestinal failure commonly includes acid suppression, antimotility agents, somatostatin analogs, and gradual advancement of enteral nutrition (12, 24, 25). These interventions are effective in reducing stoma output and stabilizing fluid balance, which may partly explain why both groups improved over time. However, despite receiving conventional supportive therapy, patients without PEC still required greater parenteral fluid and electrolyte supplementation at follow-up, suggesting an additional benefit from chyme reinfusion beyond medical management alone. We observed no significant difference in PNALD incidence between the PEC and non-PEC groups (16 vs. 3, *P* = 0.62). This may be explained by the relatively short 2-month preoperative observation period, which may be insufficient for hepatic biochemical changes to manifest after reductions in parenteral support. Longer follow-up studies are needed to determine whether PEC-guided reinfusion translates into meaningful hepatic benefits.

Several limitations should be acknowledged. First, this was a retrospective single-center study, and the non-PEC group was historical (patients treated before 2021), introducing potential selection and temporal bias. Although baseline characteristics were comparable, unmeasured confounders related to changes in perioperative care over time cannot be excluded. Second, the comparison group was notably small (*n* = 14) compared with the PEC group (*n* = 57), limiting statistical power and precision; this imbalance increases the risk of type II error. Third, the 2-month observation window may have been insufficient to capture the full nutritional impact of colonic adaptation; longer follow-up might reveal differences in BMI and albumin that were not apparent at this early time point. Fourth, patient-reported outcomes, quality of life, and cost data were not available. Fifth, detailed information on segmental colon anatomy and colonic mucosal health was lacking, precluding systematic assessment of diversion colitis as a potential outcome of colonic reactivation. Sixth, although CONUT and NRS2002 are useful screening instruments, they may not fully reflect the specific benefits of PEC in reducing parenteral support dependence and improving enteral tolerance. Seventh, urinary sodium excretion was not routinely measured in this retrospective cohort, and we relied on urine output, daily weight, and serum electrolytes to guide fluid management; we acknowledge that urinary Na >20 mEq/L would provide a more precise marker of sodium repletion and should be incorporated into future protocols. additionally, Serum D-lactate levels were not measured in this study, precluding systematic assessment of subclinical D-lactic acidosis—a theoretical metabolic risk when carbohydrate-containing chyme is reintroduced into a dormant colon. While no clinical symptoms suggestive of D-lactic acidosis were observed, we cannot exclude subclinical events. Future prospective studies should incorporate routine D-lactate monitoring to establish the complete safety profile of this intervention. Future studies should address these limitations through: (1) prospective randomized controlled trials to confirm our findings; (2) optimization of reinfusion protocols (frequency, volume, timing); (3) mechanistic studies on colonic adaptation and microbiome; (4) longer-term follow-up for reversal outcomes; (5) cost-effectiveness analyses; and (6) prospective monitoring of metabolic risks (urinary sodium, D-lactate, refeeding syndrome) and colonic mucosal health (diversion colitis).

## 5 Conclusion

In patients with type I SBS and a defunctionalized but intact colon, PEC-guided chyme reinfusion was associated with reduced parenteral fluid and electrolyte requirements, improved enteral tolerance, and increased prealbumin, while changes in global nutritional screening scores were limited within the 2-month observation window.

## Data Availability

The raw data supporting the conclusions of this article will be made available by the authors, without undue reservation.
